# Disease Modeling of Mitochondrial Cardiomyopathy Using Patient-Specific Induced Pluripotent Stem Cells

**DOI:** 10.3390/biology10100981

**Published:** 2021-09-29

**Authors:** Takeshi Tokuyama, Razan Elfadil Ahmed, Nawin Chanthra, Tatsuya Anzai, Hideki Uosaki

**Affiliations:** 1 Division of Regenerative Medicine, Center for Molecular Medicine, Jichi Medical University, Shimotsuke 329-0498, Japan; tokuyama.t@jichi.ac.jp (T.T.); d2029@jichi.ac.jp (R.E.A.); nawin2020@jichi.ac.jp (N.C.); r1204at@jichi.ac.jp (T.A.); 2 Department of Pediatrics, Jichi Medical University, Shimotsuke 329-0498, Japan

**Keywords:** mitochondrial disease, mitochondrial cardiomyopathy, induced pluripotent stem cells (iPSC), iPSC-derived cardiomyocyte

## Abstract

**Simple Summary:**

Mitochondria are essential intracellular organelles that generate energy within the cell. Mitochondria are present in all organs, and organs are powered by the energy produced by mitochondria. Mitochondria are composed of proteins encoded by nuclear and mitochondrial DNA. It is possible that mutations in nuclear and mitochondrial DNA cause alterations in proteins that make up mitochondria, resulting in mitochondrial dysfunction. Since cellular and organ functions depend on mitochondrial function, this mitochondrial dysfunction can lead to tissue dysfunction, namely mitochondrial diseases. In recent years, there have been many reports of the multifaceted functions of mitochondria. However, there is still little knowledge about the diseases. This problem arises because there is no suitable model to mimic mitochondrial diseases. In this paper, we introduce mitochondrial cardiomyopathy models that mimic patients’ cardiomyocytes using human induced pluripotent stem cells (iPSCs). The use of human iPSCs will advance the understanding of the pathogenesis of mitochondrial cardiomyopathy and the development of new drugs.

**Abstract:**

Mitochondrial cardiomyopathy (MCM) is characterized as an oxidative phosphorylation disorder of the heart. More than 100 genetic variants in nuclear or mitochondrial DNA have been associated with MCM. However, the underlying molecular mechanisms linking genetic variants to MCM are not fully understood due to the lack of appropriate cellular and animal models. Patient-specific induced pluripotent stem cell (iPSC)-derived cardiomyocytes (iPSC-CMs) provide an attractive experimental platform for modeling cardiovascular diseases and predicting drug efficacy to such diseases. Here we introduce the pathological and therapeutic studies of MCM using iPSC-CMs and discuss the questions and latest strategies for research using iPSC-CMs.

## 1. Introduction

The first report of derivation for human embryonic stem cells (ESCs) before the dawn of the new century opened a new era in medicine, especially regenerative medicine [1]. More recently, the invention of human induced pluripotent stem cells (iPSCs) [2,3,4,5] has created new opportunities to study mechanisms of human genetic diseases and develop new therapeutic strategies. The reprogramming is performed using a variety of somatic cell sources, including skin fibroblasts [6] and keratinocytes [7], peripheral blood [8,9,10], and oral mucosa [11], which exhibit different dynamics and efficiencies [12]. One area of research using human iPSCs that has received much attention is cardiology. Identification of cardiotoxicity during drug development is a major cause of failure in the development of lead compounds. Approximately 30% of drug candidates discontinue due to safety issues during their clinical development phases, with most being excluded due to cardiovascular side effects [13]. Animal models have contributed greatly to our understanding of cardiovascular diseases, but interspecies differences in genetics and physiology have prevented us from translating such findings to human therapeutics [14]. Patient-specific iPSC-derived cardiomyocytes (iPSC-CMs) provide a reliable model for the studies of human cardiomyocytes, given their shared genomic and transcriptomic profiles. Cardiac disease was the first case in which iPSCs from patients were used [15]. These models have been used to understand the pathogenesis of many inherited cardiac diseases and develop novel therapies [16,17,18,19,20,21,22,23].

Mitochondrial cardiomyopathy (MCM) is one of the cardiomyopathies associated with mitochondrial dysfunction, primarily due to mutations in genes encoding mitochondrial structure and function for cardiomyocytes. Recent studies have shown that mitochondria play important roles in heart failure, and several mitochondrial genes are involved in cardiovascular diseases [24,25,26,27,28]. However, the underlying mechanisms by which genetic variants induce MCM remain to be elucidated due to a lack of cellular and animal models.

This review will provide an overview of MCM and how mitochondrial function affects cardiac functions. Next, we will summarize the human iPSC-CM models of inherited cardiac diseases, including MCM, reported to date. Finally, we will describe the current challenges using iPSC-CM models to study cardiac diseases and provide our insights into possible solutions.

## 2. What Is Mitochondrial Cardiomyopathy (MCM)?

**Figure 1:**
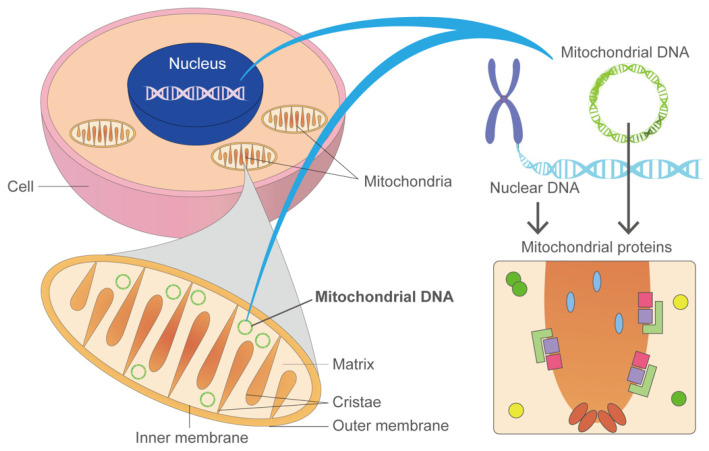
Mitochondrial diseases are caused by genetic variants in the nucleus and mitochondria DNA (nDNA and mt DNA, respectively). Most DNA is packaged in chromosomes in the nucleus, but a small amount of DNA is also present in the mitochondria. This genetic material is called mtDNA. There are 37 genes in mitochondrial DNA that are all essential for the normal functioning of mitochondria. Thirteen of these genes provide instructions for making enzymes involved in oxidative phosphorylation. Genes that cause mitochondrial diseases are found in both nDNA and mtDNA.

Mitochondria play central roles in a variety of cellular metabolic pathways, including oxidative phosphorylation, fatty acid oxidation, Krebs cycle, urea cycle, gluconeogenesis, and ketogenesis [29]. Mutations in mitochondria-related genes encoded in nuclear and mitochondrial genomes (nDNA and mtDNA, respectively) impair mitochondrial functions, resulting in many symptomatic diseases such as neuropathy (e.g., Leigh syndrome), ophthalmoplegia, diabetes, hepatopathy, nephropathy, and cardiovascular disorders [30,31] (Figure 1). These diseases are called mitochondrial diseases as a whole. Mitochondrial diseases are characterized by defects in oxidative phosphorylation (OXPHOS) [32] and are one of the largest groups of inborn errors in metabolism [33]. To date, more than 350 mitochondrial disease-related genes have been identified [34]. These disorders affect at least 1 in 5000 individuals [35]. Single genes are rarely associated with distinct clinical manifestations or modes of inheritance in mitochondrial diseases. One classic example of genetic pleiotropy in mitochondrial diseases is the mtDNA variant m.3243A > G; it is the most common pathogenic variant of *MT-TL1* and causes a wide range of phenotypes, including MELAS (mitochondrial encephalomyopathy, lactic acidosis, and stroke-like episodes), CPEO (chronic progressive external ophthalmoplegia), and MIDD (maternally inherited diabetes and deafness) [36].

**Figure 2:**
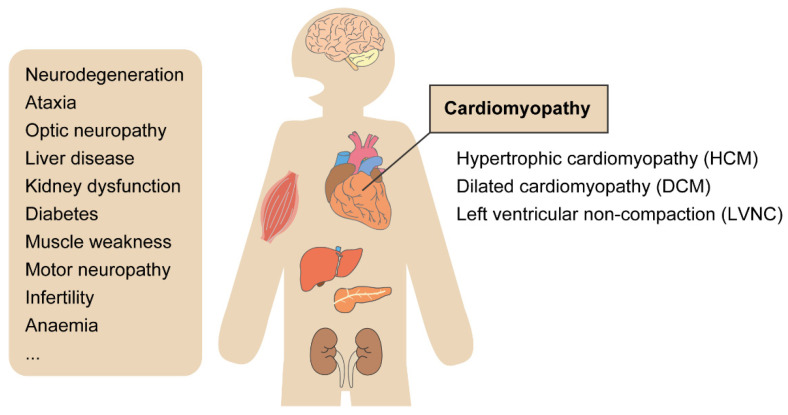
Variations in mitochondrial disease symptoms. Mitochondrial diseases can occur in children and adults and can affect a wide variety of organs, including multiple organs that have no functional connection to each other, such as the brain and liver, or beta cells of the pancreas and auditory system. Typically, these diseases are progressive. MCM could be described as a myocardial disorder characterized by abnormal cardiac-muscle structure, function, or both, secondary to genetic defects involving the mitochondrial respiratory chain. The presentations of MCMs include HCM, DCM, and LVNC, and the severity can range from no symptoms to devastating multisystemic diseases.

Mitochondrial diseases often manifest as diseases of multiple organs (Figure 2). Cardiomyopathy is common in mitochondrial diseases because the heart, such as muscle and brain, is heavily dependent on oxidative metabolism [37]. MCM is defined as a mitochondrial disease with cardiomyopathy [38]. MCM is also broadly defined as cardiomyopathy due to mitochondrial dysfunction. Cardiomyopathy has been estimated to occur in 20–40% of pediatric patients with mitochondrial diseases [39,40] and increases the mortality of the patients compared to the ones without MCM [40,41,42,43]. The combination of left ventricular hypertrophy with the neonatal onset and/or chromosomal abnormalities has an inferior prognosis. Both hypertrophic cardiomyopathy (HCM) and dilated cardiomyopathy (DCM) are typical clinical features of MCM patients. Other symptoms include arrhythmia and sudden cardiac death. In patients with Barth syndrome, an X-linked mitochondrial disease caused by variants in the *TAZ* gene, left ventricular non-compaction (LVNC) is a common heart defect [44]. MCM is broader and more complex in adults than children and involves conduction system disease, atrial fibrillation, ventricular immaturity, and ventricular preexcitation symptoms in addition to myocardial abnormalities [41].

### 2.1. Genetic Variants Associated with Mitochondrial Dysfunction

#### 2.1.1. Mitochondrial DNA Deletion

Human cells contain about 1000 mitochondria, each with two to ten copies of mitochondrial DNA (mtDNA). Human mtDNA is a 16,569 bp double-stranded circular DNA molecule and contains 37 genes encoding 13 polypeptides for OXPHOS, 22 tRNAs, and two rRNAs, essential for mitochondrial protein synthesis [45]. Point mutations in mtDNA, including small indel mutations, are a leading cause of human diseases, with an estimated prevalence of 1 in 200 in the population [46]. Clinical manifestations appear in childhood or adulthood, and variants can be inherited (~75%) or newly generated (~25%) [47]. Variants have been reported in all mtDNA genes and associated with various diseases, depending on which cells carry more mutated mtDNA [24]. A single large mtDNA deletion has a frequency of 1.5 in 100,000 in the population [48]. There are three major phenotypes of large mtDNA deletions: chronic progressive external ophthalmoplegia (~65%), Kearns-Sayre syndrome (~30%), and Pearson’s syndrome (less than 5%) [49]. Pearson’s syndrome is the most severe condition associated with a single large mtDNA deletion, with patients presenting early with sideroblastic anemia and pancreatic dysfunction, often fatal in infancy [50]. In contrast to nuclear gene rearrangements, single, large mtDNA deletions often occur sporadically during embryonic development and have a low risk of recurrence [51]. Large mtDNA deletions and point mutations are primary mtDNA defects, but secondary defects are also a common cause of mitochondrial disease. The disruption of auxiliary processes such as mtDNA maintenance, transcription, protein translation, and mitochondrial import can lead to quantitative (mtDNA copy number loss) or qualitative (mtDNA peroxidation) mtDNA defects. While some patients with mitochondrial diseases have homoplasmy in mtDNA variants (100% mutation rate), many patients have heteroplasmy, having a mixture of variant and wild-type mtDNA. The ratio of mutant mtDNA to wild-type mtDNA is important in disease manifestation, and a higher ratio of mutant mtDNA is often associated with more severe clinical symptoms. This is called a threshold effect, in which the mtDNA mutation level must exceed a critical threshold to ascertain the biochemical defect in the respiratory chain. This threshold is generally considered to be 60–80% of mtDNA but may vary across organs, variants, and individuals [52,53,54]. To note, the heteroplasmy rates across organs in a patient typically differ, which partly explains the various clinical manifestations from the same mtDNA variants.

#### 2.1.2. Variants in Nuclear and Mitochondrial DNA

Mitochondrial functions are controlled and maintained by about 1300 nuclear genes in addition to the genes on mtDNA. Most of the genes encoding the mitochondrial proteins are located in the nuclear genome and follow a Mendelian inheritance pattern [55]. Cases of de novo, X-linked, dominant, and recessive inheritance have been also reported [56,57,58,59]. Variants in more than 250 genes out of about 1300 proteins in the mitochondrial proteins are associated with mitochondrial diseases [60,61]. Several mitochondrial variants have also been associated with cardiovascular diseases [24].

The mitochondrial respiratory chain complex synthesizes adenosine triphosphate (ATP) as cellular energy [62]. Five complexes of the mitochondrial respiratory chain (complexes I, II, III, IV, and V) are embedded in the inner membrane of mitochondria. The mitochondrial respiratory chain complex enzymes are encoded in nuclear and mitochondrial genomes, and mutations in these genes cause mitochondrial dysfunction [63]. Complex I (nicotinamide adenine dinucleotide (NAD) hydrogen dehydrogenase) is composed of 44 structural subunits (7 of which are encoded by mtDNA) and at least 14 additional assembly factors [64,65]. Complex I deficiency represents approximately 30% of pediatric patients [66], of which 70–80% are caused by variants in nuclear genes [67,68]. The clinical symptoms associated with complex I deficiency are heterogeneous, although the prognosis is typically poor, with rapid progression. Succinate dehydrogenase (SDH, or complex II) is composed of four subunits (SDHA-D), all encoded by nuclear genes [69]. Heterozygous variants in *SDHB*, *SDHC*, and *SDHD* cause dominantly inherited paragangliomas and pheochromocytomas [70,71]. Among subjects with infantile mitochondrial diseases in a report, 22 of 280 (8%) patients had a complex II-specific biochemical defect [72]. Complex III comprises 11 structural subunits plus two heme groups and a Rieske (iron-sulfur) protein. Cytochrome b is the only subunit of respiratory complex III encoded in mtDNA (*MT-CYB*). More than 50% of patients with variants in the *MT-CYB* gene suffer exercise intolerance [73]. Cardiomyopathy and encephalomyopathy have also been reported in the patients [74]. Cytochrome c oxidase (COX), complex IV of the respiratory chain, is built into the inner membrane of mitochondria, functions as a dimer, and contains two copper-binding sites, two heme groups, one magnesium ion, and one zinc ion [75]. Mutations have also been reported in the structural subunits of COX, most of which affect the biosynthesis or assembly proteins [76]. Some proteins are closely associated with specific aspects of COX biosynthesis (e.g., Cytochrome c oxidase assembly factor 6, COA6, involves in copper-dependent COX2 biosynthesis [77]), while others have more diverse roles [78]. Clinically, variants in complex IV are often premature and devastating, affecting mainly the heart and central nervous system (e.g., Leigh syndrome [79]), although a mild Charcot–Marie–Tooth phenotype is also linked to two *COX6A1* variants [80]. ATP synthase (complex V) is a multimeric molecular motor that produces ATP through phosphorylation of adenosine diphosphate (ADP) using a proton driving force generated by electron transport and proton pumping by the respiratory chain. This 600 kDa complex comprises 13 subunits and is involved with at least three additional factors. Complex V deficiency due to mtDNA-encoded ATP6 (*MT-ATP6*) and ATP8 (*MT-ATP8*) genes has been reported to date [81,82,83]. Moreover, a lack of ATP synthase due to the nuclear-encoded *ATP12* and *TMEM70* genes has been reported [84,85]. The most common defect is variants in *TMEM70* that causes lactic acidosis and cardiomyopathy [84], while encephalopathy and cataract have been reported in the other populations [86]. Genetic variants are not limited to OXPHOS components but include Krebs cycle enzymes (e.g., aconitase/*ACO2* [87]) and cofactor transport (e.g., thiamine transporter/*SLC19A3* [88]). Table 1 summarizes the genes and phenotypes related to MCMs.

### 2.2. Mechanisms Linking Mitochondrial Dysfunction to Cardiac Dysfunction and the Phenotypes

Mitochondrial oxidative metabolism is a major energy source for the heart, and failure to generate or transfer energy may be the primary mechanism linking mitochondrial dysfunction to cardiac dysfunction. However, little is known about how mitochondrial dysfunction affects MCM pathogenesis. Since ATP energy is directly used for cardiomyocyte contraction, it is straightforward to hypothesize that ATP depletion causes myocardial dysfunction. However, even MCM patients, caused by mitochondrial dysfunction, have a wide variety of pathological phenotypes, and it is unlikely that mitochondrial disease mechanisms can be simply explained by ATP depletion. Mitochondria use oxygen to generate ATP as an organic fuel molecule, but they also produce reactive oxygen species (ROS) during the oxidative phosphorylation process. Although ROS have long been appreciated for their detrimental effects, there is now a greater understanding of their roles as a signaling molecule [90]. Moreover, mitochondria have been shown to maintain the intracellular environment by regulating ATP and ROS, serving as a platform for inflammation and interactions with other organelles [91,92]. Mitochondria are thus not simple energy production factories, but they regulate a variety of signals to maintain the cell. Thus, although ATP depletion is detrimental to cardiomyocytes, MCM is likely the result of a combination of a wide range of mitochondrial dysfunctions. MCM is caused by many genetic variants, and how these variants cause mitochondrial dysfunctions varies from patient to patient or variant to variant. Therefore, it is difficult to elucidate MCM pathogenesis without clarifying the effects of genetic variants one by one, and iPSC-CMs reflecting the background of each patient will be useful.

## 3. Disease Modeling with Patient-Specific iPSCs

Advances in cardiac disease research have several major limitations, including the lack of relevant tissue samples, the inability to study human cardiomyocytes longitudinally, and the lack of patient-specific drug testing platforms [93]. Traditionally, researchers have relied on cell-based assays and animal models to understand disease progression and develop therapies [94]. However, such models are known for their inability to reproducing human pathophysiology. Such models are also unable to reproduce the considerable genetic variation that exists in disease populations. These genetic variations may play a role in determining the severity of the disease and the patient’s response to drug therapy. Improved models are therefore desperately needed to understand patient-specific disease mechanisms and clinical pharmacotherapy [95]. Emerging human iPSC technology offers significant advantages over traditional models by overcoming the limitations of other human disease models. As iPSCs are a surrogate for human cardiomyocytes that is only available through biopsy and can be obtained from both healthy donors and diseased patients, they provide a powerful alternative to animals as a model for human disease. Furthermore, because iPSCs are patient-specific, they can more closely replicate the genotypes of the original donors. This could allow researchers to understand disease mechanisms at an individual patient level and to screen the efficacy and toxicity of individual drugs. For these reasons, iPSC-based models can accurately predict each patient’s unique response to various drugs, making them increasingly valuable as drug screening tools to guide clinical drug therapy [93,95,96,97].

### 3.1. Perspectives from iPSC Studies to Study Human Cells than Mice

Animal models have been extensively used to model cardiac disorders. Nonetheless, mice models need much effort for maintenance and differ from humans in many physiological aspects, which limit their ability to recapitulate human phenotypes [14]. Cardiac-specific, species-related physiological differences between humans and mice include myofilament structure, calcium handling, beating rate, critical ion channels expression, and energetics [98]. Moreover, mice models are not suitable candidates for large-scale toxicity screening and therapeutic molecules testing [99].

Although overall gene expressions are similar [100], the pattern of key myosin isoform is opposite (*Myh6* to *Myh7* in mouse hearts, *MYH7* to *MYH6* in human hearts). The expression kinetics of cardiomyocyte maturation-related genes were also different between mice and humans [101]. Some genes were expressed earlier in human hearts than mouse hearts at the corresponding stages, but others were rather expressed later. The recent transcriptome analysis revealed that the corresponding developmental ages of organs were not consistent among different species [102]. For example, mouse hearts at P0-3 and human hearts at 18–19 weeks post-conception showed the most proximity regarding the transcriptome [101]. For these reasons, mouse models have limitations to human application.

In mitochondrial diseases, a mutated mtDNA molecule and a wild-type mtDNA molecule can be present together in a single cell, which is called mtDNA heteroplasmy. Heteroplasmy ratio and variant type are key factors in determining the clinical severity of mitochondrial diseases. Furthermore, the biochemical threshold associated with the percentage of mutant mtDNA needs to be exceeded to disrupt the function of oxidative phosphorylation (OXPHOS) and develop the phenotype [103]. Reproducing mtDNA heteroplasmy in an animal model is a challenge. To date, a few mouse lines with mtDNA heteroplasmy are reported, and most of them are naturally occurred mutations rather than intended [104,105].

Patient-derived iPSCs are up for these challenges and providing a valid model of mitochondrial diseases *in vitro* as they possess pathogenic gene mutations, even if it is mtDNA heteroplasmy, chromosomal abnormality, or copy number variation, and can differentiate to many different cell types, including cardiomyocytes.

### 3.2. Cardiomyocyte Differentiation from iPSCs

Since the discovery of human ESCs and iPSCs, researchers have developed many differentiation protocols to efficiently generate cardiomyocytes with high purity [106,107,108,109]. The differentiation of cardiomyocytes from human iPSCs was first described by Zhang et al. [110]. Like ESCs, embryonic bodies were formed from human iPSCs in suspension and differentiated into cardiomyocytes, although the efficiency of this process was not sufficient [110,111,112]. Recent protocols mimic *in vivo* cardiogenesis in monolayers or suspension with the combination of recombinant growth factors, such as activin A and bone morphogenetic protein (BMP) 4, or small molecules that modulate the Wnt pathway, such as CHIR99021 and IWP2 [113,114]. In addition, metabolic selection via glucose depletion and lactate supplementation can further increase the purity of human iPSC-CMs in culture [115,116,117]. More advanced differentiation protocols aim to produce distinct atrial and ventricular populations. Recently, a method for differentiation and production of heteropolar, chamber-specific cardiac tissue with atrial and ventricular ends was reported [118].

### 3.3. Cardiac Disease Models Using Patient-Derived IPSCs

Human iPSC-CMs provide an appealing, patient-specific, infinite, and less expensive alternative to animal models [119]. The iPSC-CMs share the same genetic and molecular blueprint as the primary human cardiomyocytes, along with mechanical and electrophysiological properties. To date, a wide range of cardiac diseases have been modeled using iPSC technology, including long QT syndrome [120], Leopard syndrome [21], Brugada syndrome [121], catecholaminergic polymorphic ventricular tachycardia [122], arrhythmogenic right ventricular cardiomyopathy/dysplasia [123], DCM [124], LVNC [125], HCM [126], Andersen–Tawil syndrome [127], and Timothy syndrome [20].

### 3.4. MCM Disease Model Using Human iPSC-CMs

Recently, a few MCM disease models have been reported using iPSC-CMs. Table 2 summarizes these reports.

Barth syndrome (BTHS) is an X-linked cardiac and skeletal mitochondrial myopathy caused by variants in the *TAZ* gene encoding Tafazzin1, an acyltransferase that normally acylates cardiolipin, the major phospholipid in the mitochondrial inner membrane [134,135]. BTHS iPSC-CMs exhibit contractile dysfunction, but this is not the result of whole-cell energy depletion; rather, it is largely due to inadequate sarcomere assembly and contractile stress. ROS production was also significantly increased in BTHS iPSC-CMs, and ROS inhibition normalized the metabolism, sarcomere formation, and contractile phenotypes of BTHS iPSC-CMs. These data suggest that in BTHS iPSC-CMs, excessive ROS production contributes to sarcomere disorganization and reduced contractile stress generation [16,128,129,130].

Friedreich ataxia (FRDA) is a recessive neurodegenerative disorder associated with hypertrophic cardiomyopathy and is caused by a GAA repeat expansion in the first intron of the Frataxin (*FXN*) gene, which encodes a mitochondrial protein involved in iron-sulfur cluster biosynthesis [136,137]. Despite the presence of a disorganized mitochondrial network and reduced levels of mitochondrial DNA in FRDA iPSC-CMs, these mutated cardiomyocytes were similar to the wild-type group in terms of cell size, ATP production rate, and calcium transient properties [132,133]. However, when these cells were cultured in the presence of excess iron supplement, they exhibited hypertrophic changes, reduced ATP production, and impaired calcium handling properties. In addition, markedly enhanced iron uptake via attenuated negative feedback was also observed in FRDA iPSC-CMs. The loss of FXN and associated iron-sulfur cluster loss does not appear sufficient to alter the basal rate of energy production and calcium processing, which is an important function of cardiomyocytes [132].

Mitochondrial DNA sequencing identified a novel homoplastic 16S rRNA (*MT-RNR2*) m.2336T > C variant in a Chinese maternally inherited HCM family [138]. The m.2336T > C variant disrupts the 2336U-A2438 base pair in the stem-loop structure of 16S rRNA domain III, which is involved in the assembly of the mitochondrial ribosome. The m.2336T > C variant impairs 16S rRNA stability and mitochondrial ribosome assembly. HCM iPSC-CMs not only retained the original m.2336T > C variant but also possessed important characteristics of hypertrophied cardiomyocytes. Furthermore, the *MT-RNR2* variant increased the abundance of mitochondria to compensate for the lack of energy production [126].

Several mitochondrial disease variants have been shown using iPSC-CMs as above; however, their phenotypes are often more varied than expected. The therapeutic effects of ROS suppression have also been investigated, and research on MCM using iPSC-CMs will contribute significantly to elucidating the mechanism and treatment of the disease.

## 4. Limitations of iPSC-CMs

Although iPSC-CMs can be obtained easily and efficiently and beat consistently from a very early stage of differentiation, a few downsides exist, such as genomic instability [139] and the heterogeneity of the human iPSC-CMs populations in culture [118]. However, most importantly, iPSC-CMs are still immature and morphologically and functionally similar to fetal cardiomyocytes. Human iPSC-CMs exhibit a disorganized morphology, insufficient contractile capacity, glycolytic metabolism, and abnormal electrophysiological properties [140]. These limitations make it very difficult to model adult diseases, as it is unclear whether relatively immature iPSC-CMs can fully reproduce the adult disease phenotypes. However, there are ongoing efforts to overcome such limitations and to increase the maturity of human iPSC-CMs to an adult-like state [141]. Here, we summarize the characteristics of adult cardiomyocytes and how they differ from iPSC-CMs, and the approaches to address the issues.

### 4.1. Characteristics of Adult Cardiomyocytes and iPSC-CMs

#### 4.1.1. Morphology and Structure of Cardiomyocytes

Adult cardiomyocytes are well-aligned, rod-like, and multinucleated or tetraploid cells, with highly organized sarcomeres, well-developed sarcoplasmic reticulum (SR), and transverse tubules (T-tubules) [142,143,144], and have intercalated disks with mature mechanical and electrical junctions [119,145,146,147,148,149]. Human iPSC-CMs lack such structural maturity, resulting in small, mononuclear, more spherical cells with disorganized sarcomeres [119,142].

#### 4.1.2. Physical and Electrophysiological Properties

Adult cardiomyocytes only beat when stimulated, produce force around 40–80 mN/mm^2^, and conduct electricity at the velocity of ~60 cm/s, and their upstroke velocity of the action potential (AP) is about 150–350 V/s. These parameters are around 0.08–4 mN/mm^2^, 10–20 cm/s, and 10–50 V/s for iPSC-CMs [119]. Moreover, iPSC-CMs display mixed AP morphologies, categorized as atrial, nodal, or ventricular-like ones [147]. Although iPSC-CMs generate important cardiac currents such as I_Na_, I_Ca,L_, I_to_, I_Kr_, and I_Ks_, they lack I_K1_ essential for stabilizing the resting potential [149,150,151]. Another characteristic is the spontaneous beating of iPSC-CMs. While I_f_ currents generated by hyperpolarization-activated cyclic nucleotide-gated potassium channel 4 (HCN4) are confined to pacemaker cells *in vivo*, they depolarize membrane potential and make iPSC-CMs beat [152].

#### 4.1.3. Calcium Signaling

T-tubules and SR are well organized in mature cardiomyocytes and regulate Ca^2+^ induced Ca^2+^ release and fast excitation-contraction coupling. Ca^2+^ influx via L-type channels triggers Ca^2+^ release from SR via ryanodine-receptor channels [153]. During the relaxation phase, Ca^2+^ is returned to SR via sarco/endoplasmic reticulum Ca^2+^ ATPase (SERCA) and extruded out of the cell via Na^+^-Ca^2+^ exchanger. In iPSC-CMs, T-tubules are absent, SR is underdeveloped, and the expression of SERCA and other important proteins is low [141]. Accordingly, iPSC-CMs depend on L-type channels for Ca^2+^ rise and have slow excitation-contraction couplings [154,155].

#### 4.1.4. Metabolism

Metabolic substrate switches from glucose to fatty acids in cardiomyocytes after birth with increased mitochondrial volume and oxidative capacity changes to meet the energy demand [156]. β-oxidation of fatty acids increases as cardiomyocytes mature and becomes a major source of energy production. These metabolic changes occur soon after birth in rabbits [157]. Nevertheless, iPSC-CMs remain immature, and therefore metabolism is glycolysis-dependent rather than fatty acid β-oxidation [18,158,159,160].

#### 4.1.5. Gene Expression

The study of gene expressions involved during human cardiomyocyte maturation is still in progress. The overall expression patterns of maturation-related genes identified in mice and humans are similar, although there are some differences [100,101,161]. Isoforms of sarcomeric genes change from fetal to adult life. Troponin I (TnI) has three isoforms (slow skeleton (ssTnI), fast skeleton (fsTnI), and cardiac (cTnI)) encoded by *TNNI1*, *TNNI2*, and *TNNI3*, respectively. In iPSC-CMs, ssTnI is the major isoform, but cTnI is highly expressed in adult cardiomyocytes. There are three major isoforms of titin (*TTN*): N2B, N2BA, and fetal cardiac titin (FCT). N2B is predominantly expressed in adult cardiomyocytes, while N2BA is predominantly expressed in iPSC-CMs [119,162]. Furthermore, iPSC-CMs have lower expression levels of important cardiac genes such as *SERCA2* (sarcoplasmic reticulum ATPase 2), *CAV3* (caveolin 3), *KCNH2* (potassium potential dependent channel), and other adult cardiomyocyte genes [119,163,164]. At the myocardium level, cTnI (*TNNI3* gene product) progressively replaces ssTnI (*TNNI1*) during postnatal maturation; thus, the ratio of cTnI to ssTnI protein isoforms is a criterion for iPSC-CMs maturation status [165]. Transcriptome-based approaches such as gene regulatory network-based [166] and relative expression order-based scoring methods [167] were proposed to achieve a more accurate measurement of maturation.

### 4.2. Approaches for iPSC-CMs Maturation

The functional immaturity of iPSC-CMs has been a bottleneck in modeling and studying most cardiovascular diseases. Improving the maturity of iPSC-CM is a very important topic, and many studies have reported various methods to achieve this goal. Currently, a plethora of methods are being developed and tested to enhance iPSC-CMs maturity. These include but are not limited to prolonged culture time, hormonal cues, chemical alterations, inter-cellular interactions, biophysical properties of the culture, and the introduction of 3D culture technologies. Thyroid hormone is well known for playing an important role in cardiac development and cardiovascular physiology [168] and was shown to strongly promote the maturation of iPSC-CMs [169]. Recent works further highlighted the importance of humoral regulations [170]. The hallmark of postnatal cardiomyocyte maturation is their switch in metabolism from glycolysis to fatty acid oxidation [171]. Replacement of glucose with galactose and fatty acids (more specifically palmitic acid, oleic acid, linoleic acid, and carnitine) promoted the maturation of iPSC-CMs [158,171,172,173]. In a heart, cells interact with each other through direct cell-cell contacts and indirect paracrine factors secreted by neighboring cells, and the interactions are involved in cardiac maturation [174,175,176,177,178,179]. Cardiomyocytes are also continuously exposed to electrical stimulations and mechanical stress. Continuous electrical stimulation results in iPSC-CMs with rod-like morphology, enhanced cell alignment, and more organized sarcomeres [180]. Conventional 2D culture cannot reproduce the complex *in vivo* extracellular microenvironment. Three-dimensional culture, on the other hand, is closer to the extracellular microenvironment *in vivo*, supporting improved cell-cell interactions and allowing biochemical and physical stimuli to reach the cells evenly [181,182]. Human iPSC-CMs can mature to adult cardiomyocytes in one to two months when incubated in live rat neonatal myocardium, suggesting an appropriate microenvironment is crucial for the maturation [183]. Many researchers have devised a variety of methods to obtain mature cardiomyocytes; however, even with these developed methods, they have yet to fully mimic mature human cardiomyocytes.

The establishment of mature, adult-like iPSC-CMs will offer a promising, infinite source of patient-specific cardiomyocytes without invasive procedures to harvest primary cardiomyocytes from the patients. It will provide better, cheaper, easier to maintain, and more reliable cardiac disease modeling platforms than animal models such as mice. Moreover, since iPSC-CMs disease models can potentially recapitulate the pathophysiology of cardiac diseases, they can be used to develop further and test possible therapeutic candidates.

## 5. Future Research on MCM Using iPSC-CMs

Human iPSC-CMs offer powerful tools for drug toxicity screening, cardiovascular disease modeling, and drug discovery. Human iPSC-CMs have been used as models for several major cardiomyopathies, including ion-associated, structural, and metabolic cardiomyopathy, and have provided new insights into the mechanisms underlying the disease phenotypes [184]. The U.S. Food and Drug Administration established a new paradigm by convening a consortium of regulatory, industrial, and academic members to develop more accurate assessment techniques. This new paradigm, the comprehensive *in vitro* proarrhythmia assay (CiPA), includes preclinical assays using iPSC-CMs *in vitro* and *in silico* modeling. CiPA aims to discover the electrophysiological mechanisms underlying the proarrhythmic effects of drug candidates and is believed to be a pharmacological safety screening tool for drug development [185]. A recent international validation study of CiPA using 28 blind compounds was conducted at several centers [186], demonstrating the overall utility of iPSC-CMs. The National Institute of Health Sciences (NIHS) in Japan brought together experts from multiple disciplines to develop a new testing paradigm for predicting clinical proarrhythmic risk called Japan iPS cardiac safety assessment (JiCSA) [187,188]. Data obtained by JiCSA showed reproducible proarrhythmic risk prediction using two cell lines from different vendors [189,190]. JiCSA-selected compounds overlapped with all 28 CiPA-selected compounds. Moreover, the JiCSA data correlated well with the CiPA study, despite different analysis modalities [187]. Both CiPA and JiCSA have demonstrated the ability of iPSC-CMs to assess arrhythmia risk, which may be one of the most important applications of iPSC-CMs.

Several compounds have been tested to treat mitochondrial diseases, but not so much cardiomyopathy [191]. Human iPSC-CMs can mimic valuable patient cardiomyocytes and will help in drug discovery research for cardiomyopathy. For example, experiments with iPSC-CMs have identified abnormalities in mitochondrial and energy metabolism as the cause of trastuzumab-induced myocardial contractile dysfunction [192], and *MAP4K4* gene silencing is beneficial in the iPSC-CMs model of ischemic injury [193]. However, there is room for progress on many fronts, including increased maturity, complex cardiomyocyte subtypes, personalized medicine, scale-up production of iPSC-CMs, multiple readouts, and future applications of iPSC-CMs in the field of drug development [184]. Furthermore, based on *in vitro* and animal studies, some compounds are contraindicated against mitochondrial diseases [194,195,196]. Therefore, the choice of treatment for mitochondrial diseases should be made with caution.

Patient-specific iPSC-CMs are an ideal cell type for the pathogenesis of MCM. These cells represent a disease-affected cell type with correct oxygen metabolism and patient-specific nDNA and mtDNA. These cells can be used to identify potentially meaningful cellular phenotypes that can be used to test therapeutic candidates. We hope that this approach will lead to the discovery of curative treatments for these debilitating diseases.

## 6. Conclusions

Mitochondria are increasingly recognized as multifunctional organelles that drive ATP production and ROS leakage and as scaffolds for a range of other signals. Furthermore, mitochondrial functions are regulated by both endogenous (nDNA and mtDNA) and exogenous factors, and further research is needed to understand the pathogenesis of mitochondrial diseases. Genetic variants that cause MCMs can be categorized into several groups. However, it is still inadequate to predict symptoms by the site of a genetic variant, as genetic variants in the same molecule can cause different symptoms. The links and regulatory mechanisms between MCMs and genetic variants remain to be elucidated, and it is currently difficult to determine whether genetic variants involving mitochondria are pathogenic since the patient’s genetic background and environmental factors are involved in disease development.

In recent years, high-throughput ‘omics’ techniques capable of detecting differences in a multitude of molecular constituents in organisms (including metabolomics, proteomics, transcriptomics, genomics, and epigenomics) accompanied by sophisticated bioinformatics tools have revealed new details about mitochondrial function and dysfunctions [34]. The broad impact of mitochondria is often tested in non-cardiomyocytes. The cardiomyocytes have very different mitochondrial behavior compared to other cells [197,198]. The gene expression is different between human and mouse cardiomyocytes, making iPSC-CMs the best way to uncover human cardiomyopathies. MCM has little understanding and requires the use of model cells to elucidate the intracellular system. Human iPSC-CMs may be a suitable tool to generate a pathological model of human MCM. This could be beneficial for seeking effective treatments against mitochondrial diseases. Future approaches to these pathologies could include germline therapy and gene therapy [199]. Mitochondrial gene therapy seems to be a valuable and promising strategy to treat mitochondrial diseases [200]. These approaches may offer a reproductive option in the future to prevent mtDNA disease transmission in affected families. Currently, establishing a comprehensive pathological model of cardiomyocytes has not been completed, but the field of maturation methods is maturing with the discoveries of many researchers. Hopefully, many studies will be conducted to elucidate the pathogenesis of MCM using iPSC-CMs, leading to effective treatment, prevention, and drug discovery.

## Figures and Tables

**Table 1:** Causative genes for mitochondrial cardiomyopathy (MCMs).

a. Genes in Mitochondrial DNA to Disease Relationship for Mitochondrial Disorders			
Gene	OMIM ID	Cardiac Phenotype	Other Phenotypes/Mitochondrial Diseases
***Subunits of respiratory chain complex***			
*MT-ND1*	516000	HCM, LVNC	LHON (Leber’s hereditary optic neuropathy)
*MT-ND4*	516003	HCM	LHON, progressive dystonia
*MT-ND5*	516005	HCM. WPW	Leigh syndrome
*MT-ATP6/8*	516060	HCM	
*MT-ATP6*	516060	HCM	NARP (neurogenic muscle weakness, ataxia, and retinitis pigmentosa.), Leigh disease
*MT-ND6*	516006	DCM, HCM	LHON, MELAS (mitochondrial myopathy, encephalopathy, lactic acidosis, and stroke-like episodes)
*MT-CYB*	516020	HCM	Septo-optic dysplasia
***Mitochondrial protein synthesis***			
*MT-TL1*	590050	HCM, DCM, RCM, LVNC	MELAS, Leigh syndrome, CPEO (chronic progressive external ophthalmoplegia), mitochondrial myopathy
*MT-TI*	590045	HCM, DCM	
*MT-TK*	590060	HCM, DCM	MERRF (myoclonus epilepsy associated with ragged red fibers), Leigh syndrome
*MT-TV*	590105	HCM	Leigh syndrome
*MT-RNR1*	561000	RCM	Maternally inherited deafness
**b. Genes in Nuclear DNA to Disease Relationship for Mitochondrial Disorders**			
Gene	OMIM ID	Cardiac Phenotype	Other phenotypes/mitochondrial diseases
***Subunits of the respiratory chain complex***			
*NDUFS2*	252010	HCM	Mitochondrial complex I deficiency
*NDUFV2*	252010	HCM	Mitochondrial complex I deficiency
*NDUFA11*	252010	HCM	Mitochondrial complex I deficiency
*NDUFB11*	300403	LVNC, WPW	Mitochondrial complex I deficiency
*SDHA*	252011	DCM, LVNC	Mitochondrial complex II deficiency
***Assembly factor***			
*NDUFAF1*	252010	HCM	Mitochondrial complex I deficiency
*ACAD9*	611126	HCM	Mitochondrial complex I deficiency
*SCO2*	604377	HCM	Cytochrome c oxidase deficiency
*COX10*	220110	HCM	Mitochondrial complex IV deficiency
*COX15*	615119	HCM	Cytochrome c oxidase deficiency
*COA6*	614772	HCM	
*TMEM70*	614052	HCM	Mitochondrial complex V (ATP synthase) deficiency
***Mitochondrial protein synthesis***			
*AARS2*	614096	HCM	COXPD (combined oxidative phosphorylation deficiency) 8
*MRPS22*	611719	HCM	COXPD8
*TSFM*	610505	HCM	COXPD3
*GTPBP3*	616198	HCM, DCM	COXPD23
*MTO1*	614702	HCM	COXPD10
*ELAC2*	615440	HCM	COXPD17
***Maintenance of mitochondrial integrity***			
*TAZ*	302060	DCM, LVNC	BTHS (Barth syndrome)
*AGK*	212350	HCM	Sengers syndrome
*SLC22A5*	212140	HCM, DCM	Systemic primary carnitine deficiency
*ACADVL*	201475	HCM, DCM	Very long-chain acyl-CoA dehydrogenase (VLCAD) deficiency
*HADHA*	609015	DCM	Mitochondrial trifunctional protein (MTP) deficiency with myopathy and neuropathy
*ATAD3A-C dup*	612316	HCM	
***Mitochondrial DNA stability***			
*SLC25A4*	615418	HCM	Mitochondrial DNA depletion syndrome-12
*QRSL1*	617209	HCM	COXPD40
*KARS*	619147	HCM	Infantile-onset progressive leukoencephalopathy with or without deafness
*TOP3A*	601243	DCM	
***Iron homeostasis***			
*FXN*	229300	HCM	Friedreich ataxia
*BOLA3*	614299	HCM	Multiple mitochondrial dysfunctions syndrome-2 with hyperglycinemia
***Coenzyme Q10 biosynthesis***			
*COQ9*	614654	HCM	Coenzyme Q10 deficiency 5
*COQ4*	616276	HCM	Coenzyme Q10 deficiency 7
***Mitochondrial protein transport***			
*DNAJC19*	610198	DCM, LVNC	3-methylglutaconic aciduria type V

OMIM, Online Mendelian Inheritance in Man; DCM, dilated cardiomyopathy; HCM, hypertrophic cardiomyopathy; RCM, restrictive cardiomyopathy; LVNC, left ventricular non-compaction; WPW, Wolff–Parkinson–White syndrome. Modified from reference [43,89].

**Table 2:** List of mitochondrial diseases analyzed using iPSC-CMs.

Gene	Variants	Protein	Disease	Phenotype	Reference
*TAZ*	c.517delG	Tafazzin	Barth syndrome	Impaired sarcomere structure and function	[16,128,129,130]
	c.328T > C			Increased reactive oxygen species	
*DNAJC19*	(rs137854888)	Mitochondrial import inner membrane translocase subunit TIM14	Dilated cardiomyopathy with ataxia syndrome (DCMA)	Impaired mitochondria	[131]
				Conduction defects	

*FXN*	Expanded GAA repeats	Frataxin	Friedreich ataxia (hypertrophic cardiomyopathy)	Disorganized mitochondria	[132]
				Impaired Ca2+ handling	
				Increased BNP expression	
				Disrupted iron homeostasis	
				Mitochondrial dysfunction and degeneration	[133]
				Decreased mitochondrial membrane potential	
*MT-RNR2*	m.2336T > C	Mitochondrial encoded16S rRNA	Hypertrophic cardiomyopathy	Mitochondrial dysfunction	[126]
				decreased mitochondrial potential	
				Electrophysiological disturbances	

## Data Availability

Not applicable.

## References

[1] Thomson J.A., Itskovitz-Eldor J., Shapiro S.S., Waknitz M.A., Swiergiel J.J., Marshall V.S., Jones J.M. (1998). Embryonic stem cell lines derived from human blastocysts. Science.

[2] Yu J., Vodyanik M.A., Smuga-Otto K., Antosiewicz-Bourget J., Frane J.L., Tian S., Nie J., Jonsdottir G.A., Ruotti V., Stewart R. (2007). Induced Pluripotent Stem Cell Lines Derived from Human Somatic Cells. Science.

[3] Takahashi K., Tanabe K., Ohnuki M., Narita M., Ichisaka T., Tomoda K., Yamanaka S. (2007). Induction of pluripotent stem cells from adult human fibroblasts by defined factors. Cell.

[4] Trounson A., McDonald C. (2015). Stem Cell Therapies in Clinical Trials: Progress and Challenges. Cell Stem Cell.

[5] Kimbrel E.A., Lanza R. (2015). Current status of pluripotent stem cells: Moving the first therapies to the clinic. Nat. Rev. Drug Discov..

[6] Takahashi K., Yamanaka S. (2006). Induction of pluripotent stem cells from mouse embryonic and adult fibroblast cultures by defined factors. Cell.

[7] Aasen T., Raya A., Barrero M.J., Garreta E., Consiglio A., Gonzalez F., Vassena R., Bilić J., Pekarik V., Tiscornia G. (2008). Efficient and rapid generation of induced pluripotent stem cells from human keratinocytes. Nat. Biotechnol..

[8] Loh Y.H., Agarwal S., Park I.H., Urbach A., Huo H., Heffner G.C., Kim K., Miller J.D., Ng K., Daley G.Q. (2009). Generation of induced pluripotent stem cells from human blood. Blood.

[9] Staerk J., Dawlaty M.M., Gao Q., Maetzel D., Hanna J., Sommer C.A., Mostoslavsky G., Jaenisch R. (2010). Reprogramming of human peripheral blood cells to induced pluripotent stem cells. Cell Stem Cell.

[10] Loh Y.H., Hartung O., Li H., Guo C., Sahalie J.M., Manos P.D., Urbach A., Heffner G.C., Grskovic M., Vigneault F. (2010). Reprogramming of T cells from human peripheral blood. Cell Stem Cell.

[11] Miyoshi K., Tsuji D., Kudoh K., Satomura K., Muto T., Itoh K., Noma T. (2010). Generation of human induced pluripotent stem cells from oral mucosa. J. Biosci. Bioeng..

[12] Yamanaka S. (2009). A fresh look at iPS cells. Cell.

[13] Arrowsmith J., Miller P. (2013). Phase II and Phase III attrition rates 2011–2012. Nat. Rev. Drug. Discov..

[14] Davis R.P., van den Berg C.W., Casini S., Braam S.R., Mummery C.L. (2011). Pluripotent stem cell models of cardiac disease and their implication for drug discovery and development. Trends Mol. Med..

[15] Moretti A., Bellin M., Welling A., Jung C.B., Lam J.T., Bott-Flügel L., Dorn T., Goedel A., Höhnke C., Hofmann F. (2010). Patient-Specific Induced Pluripotent Stem-Cell Models for Long-QT Syndrome. N. Engl. J. Med..

[16] Wang G., McCain M.L., Yang L., He A., Pasqualini F.S., Agarwal A., Yuan H., Jiang D., Zhang D., Zangi L. (2014). Modeling the mitochondrial cardiomyopathy of Barth syndrome with induced pluripotent stem cell and heart-on-chip technologies. Nat. Med..

[17] Sun N., Yazawa M., Liu J., Han L., Sanchez-Freire V., Abilez O.J., Navarrete E.G., Hu S., Wang L., Lee A. (2012). Patient-Specific Induced Pluripotent Stem Cells as a Model for Familial Dilated Cardiomyopathy. Sci. Transl. Med..

[18] Kim C., Wong J., Wen J., Wang S., Wang C., Spiering S., Kan N.G., Forcales S., Puri P.L., Leone T.C. (2013). Studying arrhythmogenic right ventricular dysplasia with patient-specific iPSCs. Nature.

[19] Drawnel F.M., Boccardo S., Prummer M., Delobel F., Graff A., Weber M., Gérard R., Badi L., Kam-Thong T., Bu L. (2014). Disease Modeling and Phenotypic Drug Screening for Diabetic Cardiomyopathy using Human Induced Pluripotent Stem Cells. Cell Rep..

[20] Yazawa M., Hsueh B., Jia X., Pasca A.M., Bernstein J.A., Hallmayer J., Dolmetsch R.E. (2011). Using induced pluripotent stem cells to investigate cardiac phenotypes in Timothy syndrome. Nature.

[21] Carvajal-Vergara X., Sevilla A., D’Souza S.L., Ang Y.-S., Schaniel C., Lee D.-F., Yang L., Kaplan A.D., Adler E.D., Rozov R. (2010). Patient-specific induced pluripotent stem-cell-derived models of LEOPARD syndrome. Nature.

[22] Malan D., Zhang M., Stallmeyer B., Müller J., Fleischmann B.K., Schulze-Bahr E., Sasse P., Greber B. (2016). Human iPS cell model of type 3 long QT syndrome recapitulates drug-based phenotype correction. Basic Res. Cardiol..

[23] Itzhaki I., Maizels L., Huber I., Zwi-Dantsis L., Caspi O., Winterstern A., Feldman O., Gepstein A., Arbel G., Hammerman H. (2011). Modelling the long QT syndrome with induced pluripotent stem cells. Nature.

[24] Dominic E.A., Ramezani A., Anker S.D., Verma M., Mehta N., Rao M. (2014). Mitochondrial cytopathies and cardiovascular disease. Heart.

[25] Gustafsson A.B., Gottlieb R.A. (2008). Heart mitochondria: Gates of life and death. Cardiovasc. Res..

[26] Doenst T., Nguyen T.D., Abel E.D. (2013). Cardiac metabolism in heart failure: Implications beyond ATP production. Circ. Res..

[27] Jarreta D., Orús J., Barrientos A., Miró O., Roig E., Heras M., Moraes C.T., Cardellach F., Casademont J. (2000). Mitochondrial function in heart muscle from patients with idiopathic dilated cardiomyopathy. Cardiovasc. Res..

[28] Brown D.A., Perry J.B., Allen M.E., Sabbah H.N., Stauffer B.L., Shaikh S.R., Cleland J.G.F., Colucci W.S., Butler J., Voors A.A. (2017). Mitochondrial function as a therapeutic target in heart failure. Nat. Rev. Cardiol..

[29] Duchen M.R. (2004). Mitochondria in health and disease: Perspectives on a new mitochondrial biology. Mol. Asp. Med..

[30] Johns D.R. (1995). Mitochondrial DNA and Disease. N. Engl. J. Med..

[31] Koopman W.J.H., Willems P.H.G.M., Smeitink J.A.M. (2012). Monogenic Mitochondrial Disorders. N. Engl. J. Med..

[32] Gorman G.S., Chinnery P.F., DiMauro S., Hirano M., Koga Y., McFarland R., Suomalainen A., Thorburn D.R., Zeviani M., Turnbull D.M. (2016). Mitochondrial diseases. Nat. Rev. Dis. Primers.

[33] Wolf N.I., Smeitink J.A. (2002). Mitochondrial disorders: A proposal for consensus diagnostic criteria in infants and children. Neurology.

[34] Rahman J., Rahman S. (2018). Mitochondrial medicine in the omics era. Lancet.

[35] Elliott H.R., Samuels D.C., Eden J.A., Relton C.L., Chinnery P.F. (2008). Pathogenic mitochondrial DNA mutations are common in the general population. Am. J. Hum. Genet..

[36] Nesbitt V., Pitceathly R.D., Turnbull D.M., Taylor R.W., Sweeney M.G., Mudanohwo E.E., Rahman S., Hanna M.G., McFarland R. (2013). The UK MRC Mitochondrial Disease Patient Cohort Study: Clinical phenotypes associated with the m.3243A>G mutation--implications for diagnosis and management. J. Neurol. Neurosurg. Psychiatry.

[37] Bates M.G., Bourke J.P., Giordano C., d’Amati G., Turnbull D.M., Taylor R.W. (2012). Cardiac involvement in mitochondrial DNA disease: Clinical spectrum, diagnosis, and management. Eur. Heart J..

[38] Meyers D.E., Basha H.I., Koenig M.K. (2013). Mitochondrial cardiomyopathy: Pathophysiology, diagnosis, and management. Tex. Heart Inst. J..

[39] Honzik T., Tesarova M., Magner M., Mayr J., Jesina P., Vesela K., Wenchich L., Szentivanyi K., Hansikova H., Sperl W. (2012). Neonatal onset of mitochondrial disorders in 129 patients: Clinical and laboratory characteristics and a new approach to diagnosis. J. Inherit. Metab. Dis..

[40] Imai-Okazaki A., Kishita Y., Kohda M., Mizuno Y., Fushimi T., Matsunaga A., Yatsuka Y., Hirata T., Harashima H., Takeda A. (2019). Cardiomyopathy in children with mitochondrial disease: Prognosis and genetic background. Int. J. Cardiol..

[41] Wahbi K., Bougouin W., Béhin A., Stojkovic T., Bécane H.M., Jardel C., Berber N., Mochel F., Lombès A., Eymard B. (2015). Long-term cardiac prognosis and risk stratification in 260 adults presenting with mitochondrial diseases. Eur. Heart J..

[42] Holmgren D., Wåhlander H., Eriksson B.O., Oldfors A., Holme E., Tulinius M. (2003). Cardiomyopathy in children with mitochondrial disease; clinical course and cardiological findings. Eur. Heart J..

[43] Imai-Okazaki A., Matsunaga A., Yatsuka Y., Nitta K.R., Kishita Y., Sugiura A., Sugiyama Y., Fushimi T., Shimura M., Ichimoto K. (2021). Long-term prognosis and genetic background of cardiomyopathy in 223 pediatric mitochondrial disease patients. Int. J. Cardiol..

[44] Imai-Okazaki A., Kishita Y., Kohda M., Yatsuka Y., Hirata T., Mizuno Y., Harashima H., Hirono K., Ichida F., Noguchi A. (2018). Barth Syndrome: Different Approaches to Diagnosis. J. Pediatr..

[45] Saraste M. (1999). Oxidative phosphorylation at the fin de siècle. Science.

[46] Chinnery P.F., Elliott H.R., Hudson G., Samuels D.C., Relton C.L. (2012). Epigenetics, epidemiology and mitochondrial DNA diseases. Int. J. Epidemiol..

[47] Sallevelt S.C., de Die-Smulders C.E., Hendrickx A.T., Hellebrekers D.M., de Coo I.F., Alston C.L., Knowles C., Taylor R.W., McFarland R., Smeets H.J. (2017). De novo mtDNA point mutations are common and have a low recurrence risk. J. Med. Genet..

[48] Gorman G.S., Schaefer A.M., Ng Y., Gomez N., Blakely E.L., Alston C.L., Feeney C., Horvath R., Yu-Wai-Man P., Chinnery P.F. (2015). Prevalence of nuclear and mitochondrial DNA mutations related to adult mitochondrial disease. Ann. Neurol..

[49] Mancuso M., Orsucci D., Angelini C., Bertini E., Carelli V., Comi G.P., Donati M.A., Federico A., Minetti C., Moggio M. (2015). Redefining phenotypes associated with mitochondrial DNA single deletion. J. Neurol..

[50] Rötig A., Cormier V., Blanche S., Bonnefont J.P., Ledeist F., Romero N., Schmitz J., Rustin P., Fischer A., Saudubray J.M. (1990). Pearson’s marrow-pancreas syndrome. A multisystem mitochondrial disorder in infancy. J. Clin. Investig..

[51] Chinnery P.F., DiMauro S., Shanske S., Schon E.A., Zeviani M., Mariotti C., Carrara F., Lombes A., Laforet P., Ogier H. (2004). Risk of developing a mitochondrial DNA deletion disorder. Lancet.

[52] de Laat P., Koene S., van den Heuvel L.P., Rodenburg R.J., Janssen M.C., Smeitink J.A. (2012). Clinical features and heteroplasmy in blood, urine and saliva in 34 Dutch families carrying the m.3243A > G mutation. J. Inherit. Metab. Dis..

[53] Shoffner J.M., Lott M.T., Lezza A.M., Seibel P., Ballinger S.W., Wallace D.C. (1990). Myoclonic epilepsy and ragged-red fiber disease (MERRF) is associated with a mitochondrial DNA tRNA(Lys) mutation. Cell.

[54] White S.L., Collins V.R., Wolfe R., Cleary M.A., Shanske S., DiMauro S., Dahl H.H., Thorburn D.R. (1999). Genetic counseling and prenatal diagnosis for the mitochondrial DNA mutations at nucleotide 8993. Am. J. Hum. Genet..

[55] Calvo S.E., Clauser K.R., Mootha V.K. (2016). MitoCarta2.0: An updated inventory of mammalian mitochondrial proteins. Nucleic Acids Res..

[56] Thompson K., Majd H., Dallabona C., Reinson K., King M.S., Alston C.L., He L., Lodi T., Jones S.A., Fattal-Valevski A. (2016). Recurrent De Novo Dominant Mutations in SLC25A4 Cause Severe Early-Onset Mitochondrial Disease and Loss of Mitochondrial DNA Copy Number. Am. J. Hum. Genet..

[57] Tang S., Wang J., Lee N.C., Milone M., Halberg M.C., Schmitt E.S., Craigen W.J., Zhang W., Wong L.J. (2011). Mitochondrial DNA polymerase gamma mutations: An ever expanding molecular and clinical spectrum. J. Med. Genet..

[58] Sperl W., Fleuren L., Freisinger P., Haack T.B., Ribes A., Feichtinger R.G., Rodenburg R.J., Zimmermann F.A., Koch J., Rivera I. (2015). The spectrum of pyruvate oxidation defects in the diagnosis of mitochondrial disorders. J. Inherit. Metab. Dis..

[59] Berger I., Hershkovitz E., Shaag A., Edvardson S., Saada A., Elpeleg O. (2008). Mitochondrial complex I deficiency caused by a deleterious NDUFA11 mutation. Ann. Neurol..

[60] Mayr J.A., Haack T.B., Freisinger P., Karall D., Makowski C., Koch J., Feichtinger R.G., Zimmermann F.A., Rolinski B., Ahting U. (2015). Spectrum of combined respiratory chain defects. J. Inherit. Metab. Dis..

[61] Nouws J., Nijtmans L., Houten S.M., van den Brand M., Huynen M., Venselaar H., Hoefs S., Gloerich J., Kronick J., Hutchin T. (2010). Acyl-CoA dehydrogenase 9 is required for the biogenesis of oxidative phosphorylation complex I. Cell Metab..

[62] Leonard J.V., Schapira A.H. (2000). Mitochondrial respiratory chain disorders I: Mitochondrial DNA defects. Lancet.

[63] Kohda M., Tokuzawa Y., Kishita Y., Nyuzuki H., Moriyama Y., Mizuno Y., Hirata T., Yatsuka Y., Yamashita-Sugahara Y., Nakachi Y. (2016). A Comprehensive Genomic Analysis Reveals the Genetic Landscape of Mitochondrial Respiratory Chain Complex Deficiencies. PLoS Genet..

[64] Hirst J. (2013). Mitochondrial complex I. Annu. Rev. Biochem..

[65] Baradaran R., Berrisford J.M., Minhas G.S., Sazanov L.A. (2013). Crystal structure of the entire respiratory complex I. Nature.

[66] Kirby D.M., Crawford M., Cleary M.A., Dahl H.H., Dennett X., Thorburn D.R. (1999). Respiratory chain complex I deficiency: An underdiagnosed energy generation disorder. Neurology.

[67] Alston C.L., Howard C., Oláhová M., Hardy S.A., He L., Murray P.G., O’Sullivan S., Doherty G., Shield J.P., Hargreaves I.P. (2016). A recurrent mitochondrial p.Trp22Arg NDUFB3 variant causes a distinctive facial appearance, short stature and a mild biochemical and clinical phenotype. J. Med. Genet..

[68] Swalwell H., Kirby D.M., Blakely E.L., Mitchell A., Salemi R., Sugiana C., Compton A.G., Tucker E.J., Ke B.X., Lamont P.J. (2011). Respiratory chain complex I deficiency caused by mitochondrial DNA mutations. Eur. J. Hum. Genet..

[69] Sun F., Huo X., Zhai Y., Wang A., Xu J., Su D., Bartlam M., Rao Z. (2005). Crystal Structure of Mitochondrial Respiratory Membrane Protein Complex II. Cell.

[70] Baysal B.E. (2000). Mutations in SDHD, a Mitochondrial Complex II Gene, in Hereditary Paraganglioma. Science.

[71] Niemann S., Müller U. (2000). Mutations in SDHC cause autosomal dominant paraganglioma, type 3. Nat. Genet..

[72] Ghezzi D., Goffrini P., Uziel G., Horvath R., Klopstock T., Lochmüller H., D’Adamo P., Gasparini P., Strom T.M., Prokisch H. (2009). SDHAF1, encoding a LYR complex-II specific assembly factor, is mutated in SDH-defective infantile leukoencephalopathy. Nat. Genet..

[73] Lott M.T., Leipzig J.N., Derbeneva O., Xie H.M., Chalkia D., Sarmady M., Procaccio V., Wallace D.C. (2013). mtDNA Variation and Analysis Using Mitomap and Mitomaster. Curr. Protoc. Bioinform..

[74] Mordaunt D.A., Jolley A., Balasubramaniam S., Thorburn D.R., Mountford H.S., Compton A.G., Nicholl J., Manton N., Clark D., Bratkovic D. (2015). Phenotypic variation of TTC19-deficient mitochondrial complex III deficiency: A case report and literature review. Am. J. Med. Genet. A.

[75] Shoubridge E.A. (2001). Cytochrome c oxidase deficiency. Am. J. Med. Genet..

[76] Pitceathly R.D., Rahman S., Wedatilake Y., Polke J.M., Cirak S., Foley A.R., Sailer A., Hurles M.E., Stalker J., Hargreaves I. (2013). NDUFA4 mutations underlie dysfunction of a cytochrome c oxidase subunit linked to human neurological disease. Cell Rep..

[77] Stroud D.A., Maher M.J., Lindau C., Vögtle F.N., Frazier A.E., Surgenor E., Mountford H., Singh A.P., Bonas M., Oeljeklaus S. (2015). COA6 is a mitochondrial complex IV assembly factor critical for biogenesis of mtDNA-encoded COX2. Hum. Mol. Genet..

[78] Mourier A., Ruzzenente B., Brandt T., Kühlbrandt W., Larsson N.G. (2014). Loss of LRPPRC causes ATP synthase deficiency. Hum. Mol. Genet..

[79] Wedatilake Y., Brown R.M., McFarland R., Yaplito-Lee J., Morris A.A., Champion M., Jardine P.E., Clarke A., Thorburn D.R., Taylor R.W. (2013). SURF1 deficiency: A multi-centre natural history study. Orphanet. J. Rare. Dis..

[80] Tamiya G., Makino S., Hayashi M., Abe A., Numakura C., Ueki M., Tanaka A., Ito C., Toshimori K., Ogawa N. (2014). A mutation of COX6A1 causes a recessive axonal or mixed form of Charcot-Marie-Tooth disease. Am. J. Hum. Genet..

[81] Jonckheere A.I., Hogeveen M., Nijtmans L., van den Brand M., Janssen A., Diepstra H., van den Brandt F., van den Heuvel B., Hol F., Hofste T. (2009). A novel mitochondrial ATP8 gene mutation in a patient with apical hypertrophic cardiomyopathy and neuropathy. BMJ Case. Rep..

[82] Xu T., Pagadala V., Mueller D.M. (2015). Understanding structure, function, and mutations in the mitochondrial ATP synthase. Microb. Cell.

[83] Jonckheere A.I., Smeitink J.A., Rodenburg R.J. (2012). Mitochondrial ATP synthase: Architecture, function and pathology. J. Inherit. Metab. Dis..

[84] Cízková A., Stránecký V., Mayr J.A., Tesarová M., Havlícková V., Paul J., Ivánek R., Kuss A.W., Hansíková H., Kaplanová V. (2008). TMEM70 mutations cause isolated ATP synthase deficiency and neonatal mitochondrial encephalocardiomyopathy. Nat. Genet..

[85] De Meirleir L., Seneca S., Lissens W., De Clercq I., Eyskens F., Gerlo E., Smet J., Van Coster R. (2004). Respiratory chain complex V deficiency due to a mutation in the assembly gene ATP12. J. Med. Genet..

[86] Spiegel R., Khayat M., Shalev S.A., Horovitz Y., Mandel H., Hershkovitz E., Barghuti F., Shaag A., Saada A., Korman S.H. (2011). TMEM70 mutations are a common cause of nuclear encoded ATP synthase assembly defect: Further delineation of a new syndrome. J. Med. Genet..

[87] Metodiev M.D., Gerber S., Hubert L., Delahodde A., Chretien D., Gérard X., Amati-Bonneau P., Giacomotto M.C., Boddaert N., Kaminska A. (2014). Mutations in the tricarboxylic acid cycle enzyme, aconitase 2, cause either isolated or syndromic optic neuropathy with encephalopathy and cerebellar atrophy. J. Med. Genet..

[88] Gerards M., Kamps R., van Oevelen J., Boesten I., Jongen E., de Koning B., Scholte H.R., de Angst I., Schoonderwoerd K., Sefiani A. (2013). Exome sequencing reveals a novel Moroccan founder mutation in SLC19A3 as a new cause of early-childhood fatal Leigh syndrome. Brain.

[89] Takeda A. (2020). Mitochondrial Cardiomyopathy. J. Pediatr. Cardiol. Cardiac. Surg..

[90] Shadel G.S., Horvath T.L. (2015). Mitochondrial ROS Signaling in Organismal Homeostasis. Cell.

[91] Gordaliza-Alaguero I., Cantó C., Zorzano A. (2019). Metabolic implications of organelle-mitochondria communication. EMBO Rep..

[92] West A.P., Shadel G.S., Ghosh S. (2011). Mitochondria in innate immune responses. Nat. Rev. Immunol..

[93] Sayed N., Liu C., Wu J.C. (2016). Translation of Human-Induced Pluripotent Stem Cells: From Clinical Trial in a Dish to Precision Medicine. J. Am. Coll. Cardiol..

[94] Schwartz P.J. (1998). Do animal models have clinical value?. Am. J. Cardiol..

[95] Matsa E., Ahrens J.H., Wu J.C. (2016). Human Induced Pluripotent Stem Cells as a Platform for Personalized and Precision Cardiovascular Medicine. Physiol. Rev..

[96] Inoue H., Nagata N., Kurokawa H., Yamanaka S. (2014). iPS cells: A game changer for future medicine. EMBO J..

[97] Chen I.Y., Matsa E., Wu J.C. (2016). Induced pluripotent stem cells: At the heart of cardiovascular precision medicine. Nat. Rev. Cardiol..

[98] Altomare C., Pianezzi E., Cervio E., Bolis S., Biemmi V., Benzoni P., Camici G.G., Moccetti T., Barile L., Vassalli G. (2016). Human-induced pluripotent stem cell-derived cardiomyocytes from cardiac progenitor cells: Effects of selective ion channel blockade. Europace.

[99] Ruzzenente B., Rötig A., Metodiev M.D. (2016). Mouse models for mitochondrial diseases. Hum. Mol. Genet..

[100] Uosaki H., Taguchi Y.h. (2016). Comparative Gene Expression Analysis of Mouse and Human Cardiac Maturation. Genom. Proteom. Bioinform..

[101] Anzai T., Yamagata T., Uosaki H. (2020). Comparative Transcriptome Landscape of Mouse and Human Hearts. Front. Cell. Dev. Biol..

[102] Cardoso-Moreira M., Halbert J., Valloton D., Velten B., Chen C., Shao Y., Liechti A., Ascenção K., Rummel C., Ovchinnikova S. (2019). Gene expression across mammalian organ development. Nature.

[103] Nissanka N., Moraes C.T. (2020). Mitochondrial DNA heteroplasmy in disease and targeted nuclease-based therapeutic approaches. EMBO Rep..

[104] Lin C.S., Sharpley M.S., Fan W., Waymire K.G., Sadun A.A., Carelli V., Ross-Cisneros F.N., Baciu P., Sung E., McManus M.J. (2012). Mouse mtDNA mutant model of Leber hereditary optic neuropathy. Proc. Natl. Acad. Sci. USA.

[105] Kauppila J.H.K., Baines H.L., Bratic A., Simard M.-L., Freyer C., Mourier A., Stamp C., Filograna R., Larsson N.-G., Greaves L.C. (2016). A Phenotype-Driven Approach to Generate Mouse Models with Pathogenic mtDNA Mutations Causing Mitochondrial Disease. Cell Rep..

[106] Dunn K.K., Palecek S.P. (2018). Engineering Scalable Manufacturing of High-Quality Stem Cell-Derived Cardiomyocytes for Cardiac Tissue Repair. Front. Med..

[107] Yang L., Soonpaa M.H., Adler E.D., Roepke T.K., Kattman S.J., Kennedy M., Henckaerts E., Bonham K., Abbott G.W., Linden R.M. (2008). Human cardiovascular progenitor cells develop from a KDR+ embryonic-stem-cell-derived population. Nature.

[108] Uosaki H., Fukushima H., Takeuchi A., Matsuoka S., Nakatsuji N., Yamanaka S., Yamashita J.K. (2011). Efficient and scalable purification of cardiomyocytes from human embryonic and induced pluripotent stem cells by VCAM1 surface expression. PLoS ONE.

[109] Elliott D.A., Braam S.R., Koutsis K., Ng E.S., Jenny R., Lagerqvist E.L., Biben C., Hatzistavrou T., Hirst C.E., Yu Q.C. (2011). NKX2-5(eGFP/w) hESCs for isolation of human cardiac progenitors and cardiomyocytes. Nat. Methods.

[110] Zhang J., Wilson G.F., Soerens A.G., Koonce C.H., Yu J., Palecek S.P., Thomson J.A., Kamp T.J. (2009). Functional cardiomyocytes derived from human induced pluripotent stem cells. Circ. Res..

[111] Burridge P.W., Thompson S., Millrod M.A., Weinberg S., Yuan X., Peters A., Mahairaki V., Koliatsos V.E., Tung L., Zambidis E.T. (2011). A universal system for highly efficient cardiac differentiation of human induced pluripotent stem cells that eliminates interline variability. PLoS ONE.

[112] Pesl M., Acimovic I., Pribyl J., Hezova R., Vilotic A., Fauconnier J., Vrbsky J., Kruzliak P., Skladal P., Kara T. (2014). Forced aggregation and defined factors allow highly uniform-sized embryoid bodies and functional cardiomyocytes from human embryonic and induced pluripotent stem cells. Heart Vessels.

[113] Fonoudi H., Ansari H., Abbasalizadeh S., Larijani M.R., Kiani S., Hashemizadeh S., Zarchi A.S., Bosman A., Blue G.M., Pahlavan S. (2015). A Universal and Robust Integrated Platform for the Scalable Production of Human Cardiomyocytes From Pluripotent Stem Cells. Stem Cells Transl. Med..

[114] Kim M.S., Horst A., Blinka S., Stamm K., Mahnke D., Schuman J., Gundry R., Tomita-Mitchell A., Lough J. (2015). Activin-A and Bmp4 levels modulate cell type specification during CHIR-induced cardiomyogenesis. PLoS ONE.

[115] Tohyama S., Hattori F., Sano M., Hishiki T., Nagahata Y., Matsuura T., Hashimoto H., Suzuki T., Yamashita H., Satoh Y. (2013). Distinct Metabolic Flow Enables Large-Scale Purification of Mouse and Human Pluripotent Stem Cell-Derived Cardiomyocytes. Cell Stem Cell.

[116] Burridge P.W., Matsa E., Shukla P., Lin Z.C., Churko J.M., Ebert A.D., Lan F., Diecke S., Huber B., Mordwinkin N.M. (2014). Chemically defined generation of human cardiomyocytes. Nat. Methods.

[117] Hemmi N., Tohyama S., Nakajima K., Kanazawa H., Suzuki T., Hattori F., Seki T., Kishino Y., Hirano A., Okada M. (2014). A massive suspension culture system with metabolic purification for human pluripotent stem cell-derived cardiomyocytes. Stem. Cells Transl. Med..

[118] Zhao Y., Rafatian N., Feric N.T., Cox B.J., Aschar-Sobbi R., Wang E.Y., Aggarwal P., Zhang B., Conant G., Ronaldson-Bouchard K. (2019). A Platform for Generation of Chamber-Specific Cardiac Tissues and Disease Modeling. Cell.

[119] Denning C., Borgdorff V., Crutchley J., Firth K.S.A., George V., Kalra S., Kondrashov A., Hoang M.D., Mosqueira D., Patel A. (2016). Cardiomyocytes from human pluripotent stem cells: From laboratory curiosity to industrial biomedical platform. Biochim. Biophys. Acta (BBA.)-Mol. Cell Res..

[120] Sala L., Gnecchi M., Schwartz P.J. (2019). Long QT Syndrome Modelling with Cardiomyocytes Derived from Human-induced Pluripotent Stem Cells. Arrhythm. Electrophysiol. Rev..

[121] Liang P., Sallam K., Wu H., Li Y., Itzhaki I., Garg P., Zhang Y., Vermglinchan V., Lan F., Gu M. (2016). Patient-Specific and Genome-Edited Induced Pluripotent Stem Cell-Derived Cardiomyocytes Elucidate Single-Cell Phenotype of Brugada Syndrome. J. Am. Coll. Cardiol..

[122] Itzhaki I., Maizels L., Huber I., Gepstein A., Arbel G., Caspi O., Miller L., Belhassen B., Nof E., Glikson M. (2012). Modeling of catecholaminergic polymorphic ventricular tachycardia with patient-specific human-induced pluripotent stem cells. J. Am. Coll. Cardiol..

[123] Caspi O., Huber I., Gepstein A., Arbel G., Maizels L., Boulos M., Gepstein L. (2013). Modeling of arrhythmogenic right ventricular cardiomyopathy with human induced pluripotent stem cells. Circ. Cardiovasc. Genet..

[124] Shah D., Virtanen L., Prajapati C., Kiamehr M., Gullmets J., West G., Kreutzer J., Pekkanen-Mattila M., Heliö T., Kallio P. (2019). Modeling of LMNA-Related Dilated Cardiomyopathy Using Human Induced Pluripotent Stem Cells. Cells.

[125] Kodo K., Ong S.G., Jahanbani F., Termglinchan V., Hirono K., InanlooRahatloo K., Ebert A.D., Shukla P., Abilez O.J., Churko J.M. (2016). iPSC-derived cardiomyocytes reveal abnormal TGF-β signalling in left ventricular non-compaction cardiomyopathy. Nat. Cell. Biol..

[126] Li S., Pan H., Tan C., Sun Y., Song Y., Zhang X., Yang W., Wang X., Li D., Dai Y. (2018). Mitochondrial Dysfunctions Contribute to Hypertrophic Cardiomyopathy in Patient iPSC-Derived Cardiomyocytes with MT-RNR2 Mutation. Stem. Cell Rep..

[127] Kuroda Y., Yuasa S., Watanabe Y., Ito S., Egashira T., Seki T., Hattori T., Ohno S., Kodaira M., Suzuki T. (2017). Flecainide ameliorates arrhythmogenicity through NCX flux in Andersen-Tawil syndrome-iPS cell-derived cardiomyocytes. Biochem. Biophys. Rep..

[128] Chowdhury A., Aich A., Jain G., Wozny K., Lüchtenborg C., Hartmann M., Bernhard O., Balleiniger M., Alfar E.A., Zieseniss A. (2018). Defective Mitochondrial Cardiolipin Remodeling Dampens HIF-1α Expression in Hypoxia. Cell Rep..

[129] Liu X., Wang S., Guo X., Li Y., Ogurlu R., Lu F., Prondzynski M., Buzon S.d.l.S., Ma Q., Zhang D. (2021). Increased Reactive Oxygen Species–Mediated Ca^2+^/Calmodulin-Dependent Protein Kinase II Activation Contributes to Calcium Handling Abnormalities and Impaired Contraction in Barth Syndrome. Circulation.

[130] Fatica E.M., DeLeonibus G.A., House A., Kodger J.V., Pearce R.W., Shah R.R., Levi L., Sandlers Y. (2019). Barth Syndrome: Exploring Cardiac Metabolism with Induced Pluripotent Stem Cell-Derived Cardiomyocytes. Metabolites.

[131] Rohani L., Meng G., Machiraju P., Liu S., Wu J., Kovalchuk I., Lewis I., Shutt T., Khan A., Rancourt D. (2017). Modeling the dilated cardiomyopathy with ataxia syndrome (dcma), a pediatric mitochondrial cardiomyopathy, using cardiomyocytes derived from induced pluripotent stem cells. Can. J. Cardiol..

[132] Lee Y.-K., Ho P.W.-L., Schick R., Lau Y.-M., Lai W.-H., Zhou T., Li Y., Ng K.-M., Ho S.-L., Esteban M.A. (2014). Modeling of Friedreich ataxia-related iron overloading cardiomyopathy using patient-specific-induced pluripotent stem cells. Pflügers Arch. Eur. J. Physiol..

[133] Hick A., Wattenhofer-Donzé M., Chintawar S., Tropel P., Simard J.P., Vaucamps N., Gall D., Lambot L., André C., Reutenauer L. (2013). Neurons and cardiomyocytes derived from induced pluripotent stem cells as a model for mitochondrial defects in Friedreich’s ataxia. Dis. Models Mech..

[134] Houtkooper R.H., Turkenburg M., Poll-The B.T., Karall D., Pérez-Cerdá C., Morrone A., Malvagia S., Wanders R.J., Kulik W., Vaz F.M. (2009). The enigmatic role of tafazzin in cardiolipin metabolism. Biochim. Biophys. Acta (BBA) Biomembr..

[135] Bione S., D’Adamo P., Maestrini E., Gedeon A.K., Bolhuis P.A., Toniolo D. (1996). A novel X-linked gene, G4.5. is responsible for Barth syndrome. Nat. Genet..

[136] Schulz J.B., Boesch S., Bürk K., Dürr A., Giunti P., Mariotti C., Pousset F., Schöls L., Vankan P., Pandolfo M. (2009). Diagnosis and treatment of Friedreich ataxia: A European perspective. Nat. Rev. Neurol..

[137] Herman D., Jenssen K., Burnett R., Soragni E., Perlman S.L., Gottesfeld J.M. (2006). Histone deacetylase inhibitors reverse gene silencing in Friedreich’s ataxia. Nat. Chem. Biol..

[138] Liu Z., Song Y., Li D., He X., Li S., Wu B., Wang W., Gu S., Zhu X., Wang X. (2014). The novel mitochondrial 16S rRNA 2336T>C mutation is associated with hypertrophic cardiomyopathy. J. Med. Genet..

[139] Yoshihara M., Hayashizaki Y., Murakawa Y. (2017). Genomic Instability of iPSCs: Challenges Towards Their Clinical Applications. Stem. Cell Rev. Rep..

[140] Machiraju P., Greenway S.C. (2019). Current methods for the maturation of induced pluripotent stem cell-derived cardiomyocytes. World J. Stem Cells.

[141] Ahmed R.E., Anzai T., Chanthra N., Uosaki H. (2020). A Brief Review of Current Maturation Methods for Human Induced Pluripotent Stem Cells-Derived Cardiomyocytes. Front. Cell Dev. Biol..

[142] Yang X., Pabon L., Murry C.E. (2014). Engineering Adolescence. Circ. Res..

[143] Bergmann O., Bhardwaj R.D., Bernard S., Zdunek S., Barnabé-Heider F., Walsh S., Zupicich J., Alkass K., Buchholz B.A., Druid H. (2009). Evidence for Cardiomyocyte Renewal in Humans. Science.

[144] Peters N.S., Severs N.J., Rothery S.M., Lincoln C., Yacoub M.H., Green C.R. (1994). Spatiotemporal relation between gap junctions and fascia adherens junctions during postnatal development of human ventricular myocardium. Circulation.

[145] Vreeker A., Van Stuijvenberg L., Hund T.J., Mohler P.J., Nikkels P.G.J., Van Veen T.A.B. (2014). Assembly of the Cardiac Intercalated Disk during Pre- and Postnatal Development of the Human Heart. PLoS ONE.

[146] Kamakura T., Makiyama T., Sasaki K., Yoshida Y., Wuriyanghai Y., Chen J., Hattori T., Ohno S., Kita T., Horie M. (2013). Ultrastructural Maturation of Human-Induced Pluripotent Stem Cell-Derived Cardiomyocytes in a Long-Term Culture. Circ. J..

[147] Ma J., Guo L., Fiene S.J., Anson B.D., Thomson J.A., Kamp T.J., Kolaja K.L., Swanson B.J., January C.T. (2011). High purity human-induced pluripotent stem cell-derived cardiomyocytes: Electrophysiological properties of action potentials and ionic currents. Am. J. Physiol.-Heart Circ. Physiol..

[148] Zwi L., Caspi O., Arbel G., Huber I., Gepstein A., Park I.-H., Gepstein L. (2009). Cardiomyocyte Differentiation of Human Induced Pluripotent Stem Cells. Circulation.

[149] Dhamoon A.S., Jalife J. (2005). The inward rectifier current (IK1) controls cardiac excitability and is involved in arrhythmogenesis. Heart Rhythm..

[150] Knollmann B.C. (2013). Induced Pluripotent Stem Cell–Derived Cardiomyocytes. Circ. Res..

[151] Hoekstra M., Mummery C., Wilde A., Bezzina C., Verkerk A. (2012). Induced pluripotent stem cell derived cardiomyocytes as models for cardiac arrhythmias. Front. Physiol..

[152] Yanagi K., Takano M., Narazaki G., Uosaki H., Hoshino T., Ishii T., Misaki T., Yamashita J.K. (2007). Hyperpolarization-Activated Cyclic Nucleotide-Gated Channels and T-Type Calcium Channels Confer Automaticity of Embryonic Stem Cell-Derived Cardiomyocytes. Stem Cells.

[153] Bers D.M. (2002). Cardiac excitation–contraction coupling. Nature.

[154] Veerman C.C., Mengarelli I., Lodder E.M., Kosmidis G., Bellin M., Zhang M., Dittmann S., Guan K., Wilde A.A.M., Schulze-Bahr E. (2017). Switch From Fetal to Adult *SCN5A* Isoform in Human Induced Pluripotent Stem Cell–Derived Cardiomyocytes Unmasks the Cellular Phenotype of a Conduction Disease–Causing Mutation. J. Am. Heart Assoc..

[155] Pesl M., Pribyl J., Caluori G., Cmiel V., Acimovic I., Jelinkova S., Dvorak P., Starek Z., Skladal P., Rotrekl V. (2017). Phenotypic assays for analyses of pluripotent stem cell-derived cardiomyocytes. J. Mol. Recognit..

[156] Lopaschuk G.D., Jaswal J.S. (2010). Energy metabolic phenotype of the cardiomyocyte during development, differentiation, and postnatal maturation. J. Cardiovasc. Pharmacol..

[157] Lopaschuk G.D., Spafford M.A., Marsh D.R. (1991). Glycolysis is predominant source of myocardial ATP production immediately after birth. Am. J. Physiol.-Heart Circ. Physiol..

[158] Correia C., Koshkin A., Duarte P., Hu D., Teixeira A., Domian I., Serra M., Alves P.M. (2017). Distinct carbon sources affect structural and functional maturation of cardiomyocytes derived from human pluripotent stem cells. Sci. Rep..

[159] Kikuchi C., Bienengraeber M., Canfield S., Koopmeiner A., Schäfer R., Bosnjak Z.J., Bai X. (2015). Comparison of Cardiomyocyte Differentiation Potential between Type 1 Diabetic Donor- and Nondiabetic Donor-Derived Induced Pluripotent Stem Cells. Cell Transplant..

[160] Rana P., Anson B., Engle S., Will Y. (2012). Characterization of Human-Induced Pluripotent Stem Cell–Derived Cardiomyocytes: Bioenergetics and Utilization in Safety Screening. Toxicol. Sci..

[161] DeLaughter D.M., Bick A.G., Wakimoto H., McKean D., Gorham J.M., Kathiriya I.S., Hinson J.T., Homsy J., Gray J., Pu W. (2016). Single-Cell Resolution of Temporal Gene Expression during Heart Development. Dev. Cell.

[162] Yin Z., Ren J., Guo W. (2015). Sarcomeric protein isoform transitions in cardiac muscle: A journey to heart failure. Biochim. Biophysica. Acta (BBA) Mol. Basis Dis..

[163] Van Den Berg C.W., Okawa S., Chuva De Sousa Lopes S.M., Van Iperen L., Passier R., Braam S.R., Tertoolen L.G., Del Sol A., Davis R.P., Mummery C.L. (2015). Transcriptome of human foetal heart compared with cardiomyocytes from pluripotent stem cells. Development.

[164] Karakikes I., Ameen M., Termglinchan V., Wu J.C. (2015). Human Induced Pluripotent Stem Cell–Derived Cardiomyocytes. Circ. Res..

[165] Bedada F.B., Chan S.S.-K., Metzger S.K., Zhang L., Zhang J., Garry D.J., Kamp T.J., Kyba M., Metzger J.M. (2014). Acquisition of a Quantitative, Stoichiometrically Conserved Ratiometric Marker of Maturation Status in Stem Cell-Derived Cardiac Myocytes. Stem Cell Rep..

[166] Uosaki H., Cahan P., Lee D.I., Wang S., Miyamoto M., Fernandez L., Kass D.A., Kwon C. (2015). Transcriptional Landscape of Cardiomyocyte Maturation. Cell Rep..

[167] Chen R., He J., Wang Y., Guo Y., Zhang J., Peng L., Wang D., Lin Q., Zhang J., Guo Z. (2019). Qualitative transcriptional signatures for evaluating the maturity degree of pluripotent stem cell-derived cardiomyocytes. Stem. Cell Res. Ther..

[168] Klein I., Ojamaa K. (2001). Thyroid Hormone and the Cardiovascular System. N. Engl. J. Med..

[169] Yang X., Rodriguez M., Pabon L., Fischer K.A., Reinecke H., Regnier M., Sniadecki N.J., Ruohola-Baker H., Murry C.E. (2014). Tri-iodo-l-thyronine promotes the maturation of human cardiomyocytes-derived from induced pluripotent stem cells. J. Mol. Cell. Cardiol..

[170] Funakoshi S., Fernandes I., Mastikhina O., Wilkinson D., Tran T., Dhahri W., Mazine A., Yang D., Burnett B., Lee J. (2021). Generation of mature compact ventricular cardiomyocytes from human pluripotent stem cells. Nat. Commun..

[171] Yang X., Rodriguez M.L., Leonard A., Sun L., Fischer K.A., Wang Y., Ritterhoff J., Zhao L., Kolwicz S.C., Pabon L. (2019). Fatty Acids Enhance the Maturation of Cardiomyocytes Derived from Human Pluripotent Stem Cells. Stem Cell Rep..

[172] Horikoshi Y., Yan Y., Terashvili M., Wells C., Horikoshi H., Fujita S., Bosnjak Z.J., Bai X. (2019). Fatty Acid-Treated Induced Pluripotent Stem Cell-Derived Human Cardiomyocytes Exhibit Adult Cardiomyocyte-Like Energy Metabolism Phenotypes. Cells.

[173] Nakano H., Minami I., Braas D., Pappoe H., Wu X., Sagadevan A., Vergnes L., Fu K., Morselli M., Dunham C. (2017). Glucose inhibits cardiac muscle maturation through nucleotide biosynthesis. eLife.

[174] Yoshida S., Miyagawa S., Fukushima S., Kawamura T., Kashiyama N., Ohashi F., Toyofuku T., Toda K., Sawa Y. (2018). Maturation of Human Induced Pluripotent Stem Cell-Derived Cardiomyocytes by Soluble Factors from Human Mesenchymal Stem Cells. Mol. Ther..

[175] Talman V., Kivelä R. (2018). Cardiomyocyte—Endothelial Cell Interactions in Cardiac Remodeling and Regeneration. Front. Cardiovasc. Med..

[176] Abecasis B., Gomes-Alves P., Rosa S., Gouveia P.J., Ferreira L., Serra M., Alves P.M. (2019). Unveiling the molecular crosstalk in a human induced pluripotent stem cell-derived cardiac model. Biotechnol. Bioeng..

[177] Ogasawara T., Okano S., Ichimura H., Kadota S., Tanaka Y., Minami I., Uesugi M., Wada Y., Saito N., Okada K. (2017). Impact of extracellular matrix on engraftment and maturation of pluripotent stem cell-derived cardiomyocytes in a rat myocardial infarct model. Sci. Rep..

[178] Herron T.J., Rocha A.M.D., Campbell K.F., Ponce-Balbuena D., Willis B.C., Guerrero-Serna G., Liu Q., Klos M., Musa H., Zarzoso M. (2016). Extracellular Matrix–Mediated Maturation of Human Pluripotent Stem Cell–Derived Cardiac Monolayer Structure and Electrophysiological Function. Circ. Arrhythmia Electrophysiol..

[179] Chun Y.W., Balikov D.A., Feaster T.K., Williams C.H., Sheng C.C., Lee J.-B., Boire T.C., Neely M.D., Bellan L.M., Ess K.C. (2015). Combinatorial polymer matrices enhance in vitro maturation of human induced pluripotent stem cell-derived cardiomyocytes. Biomaterials.

[180] Chan Y.-C., Ting S., Lee Y.-K., Ng K.-M., Zhang J., Chen Z., Siu C.-W., Oh S.K.W., Tse H.-F. (2013). Electrical Stimulation Promotes Maturation of Cardiomyocytes Derived from Human Embryonic Stem Cells. J. Cardiovasc. Transl. Res..

[181] Mirbagheri M., Adibnia V., Hughes B.R., Waldman S.D., Banquy X., Hwang D.K. (2019). Advanced cell culture platforms: A growing quest for emulating natural tissues. Mater. Horiz..

[182] Duval K., Grover H., Han L.-H., Mou Y., Pegoraro A.F., Fredberg J., Chen Z. (2017). Modeling Physiological Events in 2D vs. 3D Cell Culture. Physiology.

[183] Cho G.-S., Lee D.I., Tampakakis E., Murphy S., Andersen P., Uosaki H., Chelko S., Chakir K., Hong I., Seo K. (2017). Neonatal Transplantation Confers Maturation of PSC-Derived Cardiomyocytes Conducive to Modeling Cardiomyopathy. Cell Rep..

[184] Li J., Hua Y., Miyagawa S., Zhang J., Li L., Liu L., Sawa Y. (2020). hiPSC-Derived Cardiac Tissue for Disease Modeling and Drug Discovery. Int. J. Mol. Sci..

[185] Cavero I., Holzgrefe H. (2015). CiPA: Ongoing testing, future qualification procedures, and pending issues. J. Pharmacol. Toxicol. Methods.

[186] Blinova K., Dang Q., Millard D., Smith G., Pierson J., Guo L., Brock M., Lu H.R., Kraushaar U., Zeng H. (2018). International Multisite Study of Human-Induced Pluripotent Stem Cell-Derived Cardiomyocytes for Drug Proarrhythmic Potential Assessment. Cell Rep..

[187] Kanda Y., Yamazaki D., Osada T., Yoshinaga T., Sawada K. (2018). Development of torsadogenic risk assessment using human induced pluripotent stem cell-derived cardiomyocytes: Japan iPS Cardiac Safety Assessment (JiCSA) update. J. Pharmacol. Sci..

[188] Kanda Y., Yamazaki D., Kurokawa J., Inutsuka T., Sekino Y. (2016). Points to consider for a validation study of iPS cell-derived cardiomyocytes using a multi-electrode array system. J. Pharmacol. Toxicol. Methods..

[189] Ando H., Yoshinaga T., Yamamoto W., Asakura K., Uda T., Taniguchi T., Ojima A., Shinkyo R., Kikuchi K., Osada T. (2017). A new paradigm for drug-induced torsadogenic risk assessment using human iPS cell-derived cardiomyocytes. J. Pharmacol. Toxicol. Methods..

[190] Yamazaki D., Kitaguchi T., Ishimura M., Taniguchi T., Yamanishi A., Saji D., Takahashi E., Oguchi M., Moriyama Y., Maeda S. (2018). Proarrhythmia risk prediction using human induced pluripotent stem cell-derived cardiomyocytes. J. Pharmacol. Sci..

[191] Weissig V. (2020). Drug Development for the Therapy of Mitochondrial Diseases. Trends Mol. Med..

[192] Kitani T., Ong S.G., Lam C.K., Rhee J.W., Zhang J.Z., Oikonomopoulos A., Ma N., Tian L., Lee J., Telli M.L. (2019). Human-Induced Pluripotent Stem Cell Model of Trastuzumab-Induced Cardiac Dysfunction in Patients With Breast Cancer. Circulation.

[193] Fiedler L.R., Chapman K., Xie M., Maifoshie E., Jenkins M., Golforoush P.A., Bellahcene M., Noseda M., Faust D., Jarvis A. (2019). MAP4K4 Inhibition Promotes Survival of Human Stem Cell-Derived Cardiomyocytes and Reduces Infarct Size In Vivo. Cell Stem Cell.

[194] Hargreaves I.P., Al Shahrani M., Wainwright L., Heales S.J. (2016). Drug-Induced Mitochondrial Toxicity. Drug Saf..

[195] Morén C., Juárez-Flores D.L., Cardellach F., Garrabou G. (2016). The Role of Therapeutic Drugs on Acquired Mitochondrial Toxicity. Curr. Drug Metab..

[196] Finsterer J., Segall L. (2010). Drugs interfering with mitochondrial disorders. Drug Chem. Toxicol..

[197] Song M., Dorn G.W. (2015). Mitoconfusion: Noncanonical Functioning of Dynamism Factors in Static Mitochondria of the Heart. Cell Metab..

[198] Liao H., Qi Y., Ye Y., Yue P., Zhang D., Li Y. (2021). Mechanotranduction Pathways in the Regulation of Mitochondrial Homeostasis in Cardiomyocytes. Front. Cell Dev. Biol..

[199] Mancuso M., Orsucci D., Filosto M., Simoncini C., Siciliano G. (2012). Drugs and mitochondrial diseases: 40 queries and answers. Expert Opin. Pharmacother..

[200] Coutinho E., Batista C., Sousa F., Queiroz J., Costa D. (2017). Mitochondrial Gene Therapy: Advances in Mitochondrial Gene Cloning, Plasmid Production, and Nanosystems Targeted to Mitochondria. Mol. Pharm..

